# Impact of displacement context on psychological distress in refugees resettled in Australia: a longitudinal population-based study

**DOI:** 10.1017/S2045796022000324

**Published:** 2022-07-12

**Authors:** Angela Nickerson, Shraddha Kashyap, David Keegan, Ben Edwards, Walter Forrest, Richard A. Bryant, Meaghan O'Donnell, Kim Felmingham, Alexander C. McFarlane, Wietse A. Tol, Lonneke Lenferink, Joel Hoffman, Belinda J. Liddell

**Affiliations:** 1 School of Psychology, University of New South Wales, Sydney, Australia; 2 HOST International, Sydney, Australia; 3 Centre for Social Research, The Australian National University, Canberra, Australia; 4 School of Law, University of Limerick, Ireland; 5 Department of Psychiatry, University of Melbourne, Parkville, Victoria, Australia; 6 Phoenix Australia: Centre for Posttraumatic Mental Health, Carlton, Victoria, Australia; 7 School of Psychological Sciences, University of Melbourne, Melbourne, Victoria, Australia; 8 The Centre for Traumatic Stress, University of Adelaide, Adelaide, South Australia, Australia; 9 Department of Public Health, University of Copenhagen, Copenhagen, Denmark; 10 University of Groningen: Rijksuniversiteit Groningen, Groningen, Netherlands

**Keywords:** Displaced, human-caused trauma, refugee camp, refugees, settlement, social determinants, torture, urban displacement, war/armed conflict as civilian

## Abstract

**Aims:**

Refugees typically spend years in a state of protracted displacement prior to permanent resettlement. Little is known about how various prior displacement contexts influence long-term mental health in resettled refugees. In this study, we aimed to determine whether having lived in refugee camps *v.* community settings prior to resettlement impacted the course of refugees' psychological distress over the 4 years following arrival in Australia.

**Methods:**

Participants were 1887 refugees who had taken part in the Building a New Life in Australia study, which comprised of five annual face-to-face or telephone surveys from the year of first arrival in Australia.

**Results:**

Latent growth curve modelling revealed that refugees who had lived in camps showed greater initial psychological distress (as indexed by the K6) and faster decreases in psychological distress in the 4 years after resettling in Australia, compared to those who had lived in community settings. Investigation of refugee camp characteristics revealed that poorer access to services in camps was associated with greater initial distress after resettlement, and greater ability to meet one's basic needs in camps was associated with faster decreases in psychological distress over time.

**Conclusions:**

These findings highlight the importance of the displacement context in influencing the course of post-resettlement mental health. Increasing available services and meeting basic needs in the displacement environment may promote better mental health outcomes in resettled refugees.

The United Nations High Commissioner for Refugees (UNHCR) estimates that there are currently over 82 million refugees, asylum-seekers and other displaced persons worldwide (we use the term ‘refugees' in this article for parsimony; UNHCR, 2021). Rates of mental disorders in refugees are substantially higher than the general population in host countries (Charlson *et al*., 2019). While many refugees spend years in exile after fleeing their home countries, relatively little is known about the association between transitional displacement contexts and mental health in resettled refugees.

During displacement, refugees live in highly varied contexts. Approximately 20% of refugees live in refugee camps run by the UNHCR or another non-government organisation, while 80% live in urban or rural community settings (USA for UNHCR, 2020). These contexts differ markedly in terms of the supports provided to refugees. For example, in camp environments, refugees may be provided with supports for basic needs such as food, water, shelter and healthcare (with the extent of these supports being influenced by humanitarian needs and funding) (Bakewell, 2003; Wright and Plasterer, 2010; UNHCR, 2014; SPHERE, 2018). In contrast, in community settings (particularly in low and middle-income countries or in countries that are not signatories to United Nations agreements relating to refugees), refugees are often responsible for meeting their own basic needs without legal status, work rights or access to financial assistance or formal support (Campbell, 2006; Thomas *et al*., 2011; Crisp *et al*., 2012; Zetter and Ruadel, 2016; Im *et al*., 2017; Wachter *et al*., 2018; Logie *et al*., 2019; Kunpeuk *et al*., 2021). Stressors encountered in camp and community contexts may also differ considerably. For example, in refugee camps, individuals may experience restricted movement and economic opportunities, as well as overcrowding and high rates of gender-based violence. In contrast, while refugees in community settings may have greater freedom of movement and opportunity for agency, they may be vulnerable to discrimination, violence, arrests and exploitation (Campbell, 2006; Thomas *et al*., 2011; Crisp *et al*., 2012; Zetter and Ruadel, 2016; Im *et al*., 2017; Wachter *et al*., 2018; Logie *et al*., 2019; Kunpeuk *et al*., 2021).

Understanding how different displacement contexts influence the course of psychological distress in resettled refugees is an outstanding research question. The majority of research on resettled refugees' mental health groups all pre-resettlement factors into one pre-resettlement risk domain, with few studies examining how various protracted, transitional displacement contexts may influence mental health after resettlement. Increased knowledge regarding how displacement experiences impact psychological functioning would guide service providers and policy-makers to target mental health and psychosocial support for refugees across different phases of displacement, and to tailor this support over time to improve refugee adaptation

Accordingly, in this population-based study, we proposed to investigate whether displacement context (camp *v.* community environment) was associated with different patterns in psychological distress for refugees both upon arrival and over a 4-year settlement period in Australia. We also examined whether specific characteristics of refugee camps were associated with the course of psychological distress over 4 years after settlement in Australia.

## Methods

### Participants

Participants were 1887 refugees who were resettled in Australia, and who participated in the Building a New Life in Australia (BNLA) study. The BNLA study represents a population-based cohort study, investigating resettlement experiences of refugees in Australia. The BNLA study was conducted by the Australian Department of Social Services (DSS), and the Australian Institute of Family Studies (Edwards *et al*., 2017). Data were collected annually in five waves (October 2013 to February 2018). See online Supplementary materials for detailed information regarding recruitment.

Participants in this study were restricted to those who had come to Australia via an offshore resettlement pathway (e.g. had been granted refugee status prior to arriving in Australia), with participants having come to Australia via an onshore resettlement pathway (e.g. seeking asylum after arrival in Australia) being excluded. This was because the experiences of these two groups were markedly different. For example, participants who had travelled to Australia via an onshore resettlement pathway had often endured perilous journeys by boat and immigration detention, and then held insecure visa status, in contrast to those who travelled via an offshore resettlement pathway who received refugee status while living overseas, travelled by plane and immediately settled in the community after arrival. Given the central research question of this study was to investigate whether having previously lived in a refugee camp influenced subsequent mental health, and the sample size of onshore participants was too small to undertake a separate analysis (367 individuals who travelled via an onshore pathway overall, with 28 having lived in a refugee camp), they were excluded from this study.

### Measures

Detailed information regarding measures is presented in online Supplementary materials. We measured *demographic factors* (including age, gender, education, region of origin), as well as the number of pre-resettlement *potentially traumatic events* (PTEs) participants had experienced. In terms of *pre-settlement context*, participants indicated whether they had ever spent time in a refugee camp (Yes/No) before they came to Australia, using the following definition of a refugee camp: ‘There are many refugee camps around the world. They are usually run by the United Nations and often house thousands of refugees who are waiting for their claims to be processed by the UNHCR’. In relation to the pre-settlement context variable, it is important to consider how participants defined refugee camps when answering this question. For example, while 29% of the sample reported having lived in refugee camps in Indonesia, however there are no formal refugee camps run by the Indonesian government or UNHCR in this country (Missbach, 2017). In this case, it is likely that these participants were referring to shelters overseen by the International Organization of Migration where many refugees were housed at the time of the study, focused on meeting basic needs of refugees who were not able to access government assistance due to lack of legal status. Such contexts share similarities with camps, such that residents are usually provided with supports in meeting basic needs, and certain freedoms (such as movement) may be restricted. Accordingly, in the current study, findings relating to refugee camp status these results should be considered in the context of camp-like environments, although we refer to these settings as ‘refugee camps’ for parsimony.

Characteristics of refugee camps measured in this study included: the *number of refugee camps* participants had resided in (ranging from 1 to 4), the *location of refugee camps*, *how long* participants had resided in refugee camps overall and whether participants had *family with them in the camp* (yes/no). *Poor access to services* in refugee camps was measured using a count of types of services (health/medical, school, English language classes, job training, employment, counselling, legal) participants *did not* have access to in the camp. *Meeting of basic needs* was measured using a mean score of how frequently participants had access to six types of resources or facilities in refugee camps (adequate shelter, enough food, clean water, felt safe, stayed physically healthy, feel you could practice culture/religion), with the response scale ranging from 1 = never to 4 = always. (While there is some conceptual overlap between poor access to services and meeting one's basic needs, these are considered distinct constructs whereby the first addresses the extent to which external services were available to the individual, and the latter refers to the extent to which an individual is able to have his/her basic needs met. The distinction between these two constructs is represented by a non-significant, small-to-moderate correlation between the scales (*r* = −0.13, *p* = 0.061).) *Psychological distress* was assessed using a total score from the Kessler-6 Scale (Kessler *et al*., 2010) which measures anxiety and depression symptoms over the past 4 weeks. *Perceived stressors* were measured at baseline using a count variable of stressors including work, housing, finances, school/study, caring for family, family's safety, loneliness, language barriers, discrimination, getting used to life in Australia and worrying about friends/family overseas.

### Procedure

Data for waves 1, 3 and 5 were collected *via* computer-assisted self-interview software, or *via* a computer-assisted personal interview during home visits that took place within 3–6 months after resettlement across 11 cities, and regional areas within Australia (Edwards *et al*., 2017). Data for waves 2 and 4 were collected *via* a telephone interview. The strategy of alternating home visits and telephone interviews was implemented from the inception of the project. All interviews were usually conducted with native bilingual language speakers; however, participants could also choose to complete the survey with the help of accredited interpreters. Interviews lasted between 20 min and 1 h and questions were translated into nine languages: Arabic, Burmese, Dari, Hazaragi, Persian, Chin Haka, Nepali, Swahili and Tamil. All questionnaire and interview material underwent a rigorous translation and quality assurance process, including multiple stages of independent checking.

Ethics approval for this study was obtained from the Australian Institute of Family Studies Human Research Ethics Committee (Ref# 13/03). The authors assert that all procedures contributing to this work comply with the ethical standards of the relevant national and institutional committees on human experimentation and with the Helsinki Declaration of 1975, as revised in 2013. For a more detailed review of the study procedure, refer to the data user's guide (Australian Department of Social Services, 2018).

#### Data analysis

First, we conducted a logistic regression analysis where camp status (previously lived in camp or not) was modelled as the outcome variable, and predictors were participant characteristics. This was to identify confounding factors that should be controlled for in subsequent investigations. We then conducted latent growth curve modelling (LGCM) using MPlus 8 (Muthen and Muthen, 1998–2019) to investigate overall change in psychological distress (represented by scores on the K6 at waves 1–5) over 4 years for refugees resettled in Australia, accounting for household clustering, before examining whether camp characteristics (and other variables) differentially predicted baseline psychological distress and change in psychological distress over time. Time was parameterised as 0, 1, 2, 3 and 4. We estimated models using a maximum likelihood estimator with robust standard errors. Model fit was evaluated using the Akaike information criterion (AIC), Bayesian information criterion (BIC) (with lower values representing better fit), the comparative fit index (CFI), Tucker–Lewis index (TLI) (values >0.95) and the root mean square error of approximation (RMSEA) (values <0.06) (Hu and Bentler, 1999). Next, we examined the extent to which covariates representing characteristics and experiences prior to arrival in Australia predicted intercept and slope. Multiple imputation (20 datasets) was used to account for missing data on covariates. Next, we investigated whether particular characteristics or experiences in refugee camps influenced the course of psychological distress in the 4 years after resettlement in Australia for the 381 participants who indicated they had previously resided in 1+ refugee camps. (Characteristics of community settings were not measured in this study.) These analyses were analogous to those described above conducted with the entire sample. For a detailed description of data analysis methods see online Supplementary materials.

## Results

### Participant characteristics

Participant characteristics are presented in Table 1. Nearly one-fifth of the sample (*n* = 381, 19.0%) had previously lived in a refugee camp. Logistic regression analyses predicting camp status are presented in Table 2, with the model fitting the data well: *χ*^2^(8) = 469.08, *p* < 0.001. Significant predictors of having previously lived in a refugee camp included younger age, male gender, lower likelihood of tertiary education, lower likelihood of originating from North Africa/Middle East and Asia than other countries (which were predominantly in East/Central Africa and Oceania), and lower PTE exposure prior to resettlement. Participants who had lived in refugee camps reported significantly greater perceived stressors at baseline than those who had lived in non-camp environments, but significantly lower perceived stressors at waves 2–4 (see online Supplementary materials).

**Table 1.** Characteristics of overall sample, and participants who had and had not previously lived in a refugee camp

	Overall *n* = 1887		Camp *n* = 381		Community settings *n* = 1506	
	*N*/mean	%/s.d.	*N*/mean	%/s.d.	*N*/mean	%/s.d.
Age	37.24	13.87	34.61	12.74	38.08	14.08
Gender (female)	936	49.6%	135	35.40%	787	53.30%
Highest completed education						
None	333	17.8%	106	28.00%	224	15.30%
Primary	379	20.3%	91	24.10%	283	19.30%
Secondary	883	47.2%	162	42.90%	707	48.30%
Tertiary	275	14.7%	19	5.00%	251	17.10%
Region of origin						
North Africa/Middle East	1091	57.8%	54	14.20%	1022	69.20%
South East Asia/North-East Asia	129	8.8%	20	5.2	101	6.80%
Southern/Central Asia	582	30.8%	251	65.90%	325	22.00%
Sub-Saharan Africa	85	4.5%	56	14.70%	28	1.90%
Country of birth						
Iraq	869	46.1%	10	2.60%	845	57.20%
Afghanistan	445	23.6%	135	35.40%	309	20.90%
Iran	181	9.6%	37	9.70%	143	9.70%
Myanmar	129	6.8%	20	5.20%	101	6.80%
Bhutan	84	4.5%	80	21.00%	1	0.10%
Pakistan	65	2.9%	1	0.30%	10	0.70%
Democratic Republic of Congo	35	1.9%	34	8.90%	1	0.10%
Sri Lanka	21	1.1%	18	4.70%	3	0.20%
Ethiopia	21	1.1%	5	1.30%	15	1.00%
Syria	20	1.1%	0	0.00%	20	1.40%
Eritrea	15	0.8%	6	1.60%	9	0.60%
Sudan	13	0.7%	7	1.80%	6	0.40%
Nepal	12	0.6%	12	3.10%	0	0.00%
Egypt	8	0.4%	0	0.00%	8	0.20%
India	8	0.4%	5	1.30%	2	0.10%
PTE exposure	1.87	1.30	1.64	1.43	1.94	3.02
Lived in camp	381	19.0%	–		–	

s.d., standard deviation

**Table 2.** Logistic regression predicting camp status

	*B*	s.e.	Wald	*p*	Odds ratio	95% CI Lo	95% CI Hi
Age	−0.02	0.01	6.21	0.013	0.99	0.97	1.00
Gender (female)	−1.04	0.15	49.29	<0.001	0.36	0.27	0.47
Highest completed education							
None	–	–	–	–	–	–	–
Primary	−0.07	0.20	0.43	0.513	0.94	0.63	1.39
Secondary	−0.37	0.20	3.60	0.058	0.69	0.47	1.02
Tertiary	−0.99	0.33	9.51	0.002	0.37	0.20	0.71
Region of origin							
East/Central Africa/Oceania	–	–	–	–	–	–	–
North Africa/Middle East	−3.83	0.29	174.33	<0.001	0.02	0.01	0.04
Asia	−1.55	0.27	28.67	<0.001	0.21	0.12	0.36
PTE exposure	−0.12	0.05	11.59	<0.001	0.89	0.80	0.98

B, unstandardized coefficient; s.d., standard deviation; 95% CI, 95% confidence interval.

### Camp characteristics

Characteristics of camp experiences for refugees who had previously lived in camps are presented in Table 3. It is notable that over three-quarters of the sample had lived in a single refugee camp, with approximately half of participants reporting having lived in a camp in Indonesia at some point, reflecting the pathway through Indonesia to Australia for many refugees. Over three-quarters of the sample had lived in a refugee camp for longer than 3 years, and approximately half had had at least one family member with them in the camp.

**Table 3.** Refugee camp characteristics

	*N*/mean	%/s.d.
Number of refugee camps		
1	298	78.2%
2	61	16.0%
3	17	4.5%
4	5	1.3%
Camp location		
Indonesia	109	28.6%
Nepal	41	10.7%
Iran	36	9.4%
Malawi	20	5.2%
Time spent in camps		
<1 year	26	6.8%
1–2 years	9	2.4%
3–10 years	125	32.8%
10+ years	87	22.8%
Family in camp	131	34.4%
Wife/husband	71	18.6%
Child	85	22.3%
Parent	44	11.5%
Brother/sister	72	18.9%
Grandparent	17	4.5%
Aunt/uncle	30	7.9%
Cousin	34	8.9%
Number of family in camp	2.69	1.86
Poor access to camp services (mean)	2.15	2.06
Lack of access to: health/medical	153	40.2%
School	101	26.5%
English language classes	139	36.5%
Job training	130	34.1%
Employment	125	32.8%
Counselling	71	18.6%
Legal services	69	18.1%
Other	27	7.1%
Ability to meet basic needs (mean)	2.63	0.73

s.d., standard deviation

### Overall sample

#### Change in psychological distress over time in overall sample

Mean scores on the K6 at each time-point were as follows: T1 mean = 13.34, T2 mean = 12.88, T3 mean = 13.58, T4 mean = 11.93, T5 mean = 12.83. LGCM analyses of changes in psychological distress over time in the overall sample suggested that the model comprising intercept and linear slope (AIC = 48 578, BIC = 48 633, RMSEA = 0.06, CFI = 0.95, TLI = 0.95), better fit the data than the intercept-only model (AIC = 48 658, BIC = 48 697, RMSEA = 0.07, CFI = 0.90, TLI = 0.92). Compared to the linear model, the quadratic model (AIC = 48 569, BIC = 48 647, RMSEA = 0.08, CFI = 0.95, TLI = 0.92) showed a small decrease in AIC and improvement in CFI, but evidenced an increase in BIC and a decrease in TLI. Accordingly, the linear-only model was retained for parsimony. This decision was supported by the finding that the mean of the quadratic slope was not statistically significant in the quadratic model. The linear slope model showed a significant intercept (*B* = 13.29, s.e. = 0.15, *β* = 3.24, *p* < 0.001) and negative slope (*B* = −0.22, s.e. = 0.05, *β* = −0.25, *p* < 0.001) suggesting respectively that psychological distress was significantly greater than zero at baseline for this sample, and that psychological distress decreased overall over the period of 4 years. There was a significant negative association between intercept and slope (*β* = −0.30, *p* < 0.001), suggesting that those with higher baseline distress showed greater decreases in distress over time.

#### Predictors of baseline psychological distress and change in psychological distress in overall sample

Having previously lived in a refugee camp was associated with greater baseline psychological distress and greater reductions in psychological distress over time (Table 4). Female gender, older age and greater PTE exposure, and being from Middle East/North Africa or Asia were associated with greater psychological distress at baseline. Tertiary education (compared to no education) and being from the Middle East/North Africa or Asia were associated with greater reductions in psychological distress over time.

**Table 4.** Predictors of baseline psychological distress and change in psychological distress over time in overall sample

	*B*	s.e.	*β*	*t*	*p*
Baseline psychological distress					
Previously lived in camp	0.86	0.33	0.08	2.62	0.009
Gender	1.52	0.23	0.19	6.71	<0.001
Age	0.05	0.01	0.18	5.13	<0.001
Education					
No education	–	–	–	–	–
Primary education	−0.55	0.41	−0.05	−1.33	0.185
High school education	−0.45	0.39	−0.05	−1.13	0.257
Tertiary education	−0.08	0.53	−0.01	−0.15	0.885
Region of origin					
Sub-Saharan Africa	–	–	–	–	–
Middle East/North Africa	3.78	0.61	0.46	6.22	<0.001
Asia	1.25	0.60	0.15	2.07	0.038
PTE exposure	0.29	0.11	0.09	2.78	0.005
Change in psychological distress					
Previously lived in camp	−0.64	0.12	−0.30	−5.20	<0.001
Gender	−0.04	0.08	−0.02	−0.53	0.593
Age	0.01	0.01	0.04	0.69	0.493
Education					
No education	–	–	–	–	–
Primary education	0.08	0.15	0.04	0.55	0.582
High school education	−0.07	0.14	−0.04	−0.50	0.621
Tertiary education	−0.47	0.18	−0.19	−2.69	0.007
Region of origin					
East/Central Africa/Oceania	–	–	–	–	–
North Africa/Middle East	−0.68	0.21	−0.38	−3.21	0.001
Asia	−0.61	0.21	−0.34	−2.96	0.003
PTE exposure	0.01	0.04	0.01	0.18	0.857

#### Camp characteristics predicting baseline psychological distress and change in psychological distress in participants who had lived in camps

LGCM analyses of changes in psychological distress over time in participants who had previously lived in refugee camps indicated that the model comprises intercept and linear slope best fit the data (Table 5) (AIC = 9332, BIC = 9361, RMSEA ≤ 0.001, CFI = 1.00, TLI = 1.00). This linear slope model showed a significant intercept (*B* = 12.30, s.e. = 0.27, *β* = 4.31, *p* < 0.001) and negative slope (*B* = −0.59, s.e. = 0.10, *β* = −0.86, *p* < 0.001) suggesting that psychological distress was significantly greater than zero at baseline for this sample, and that, psychological distress decreased overall over the period of 4 years. There was no significant negative association between intercept and slope, suggesting that this finding was not due to regression to the mean (see online Supplementary materials for full results).

**Table 5.** Predictors of baseline psychological distress and change in psychological distress over time in participants who had resided in refugee camps (*n* = 381)

	*B*	s.e.	*β*	*t*	*p*
*Baseline psychological distress*					
Age	0.05	0.02	0.24	2.38	0.017
Gender (female)	1.38	0.67	0.24	2.07	0.038
Camp location					
Indonesia	−0.25	1.03	−0.04	−0.24	0.812
Iran	0.17	1.03	0.02	0.16	0.873
Nepal	1.58	1.06	0.21	1.49	0.137
Malawi	0.44	1.13	0.05	0.39	0.695
Number of camps	0.13	0.44	0.03	0.30	0.796
Time in camp					
<1 year					
1–2 years	−0.23	1.27	−0.01	−0.18	0.856
3–5 years	−0.63	1.04	−0.11	−0.60	0.547
5+ years	0.08	0.77	0.02	0.11	0.914
Had family in camp	0.73	0.85	0.13	0.86	0.392
Poor access to camp services	0.26	0.13	0.19	1978	0.048
Ability to meet basic needs	0.04	0.42	0.01	0.10	0.918
*Change in psychological distress*					
Age	<0.01	0.01	0.04	0.31	0.801
Gender (female)	0.10	0.23	0.07	0.47	0.672
Camp location					
Indonesia	0.22	0.40	0.16	0.63	0.577
Iran	0.51	0.44	0.26	1.17	0.239
Nepal	−0.38	0.39	−0.21	−0.96	0.329
Malawi	0.18	0.43	0.08	0.44	0.667
Number of camps	0.21	0.18	0.19	1.10	0.244
Time in camp					
<1 year					
1–2 years	−1.05	0.48	−0.24	−2.26	0.029
3–5 years	−0.25	0.42	−0.17	−0.62	0.559
5+ years	−0.18	0.32	−0.13	−0.67	0.569
Had family in camp	−0.11	0.27	−0.08	−0.36	0.687
Poor access to camp services	−0.06	0.05	−0.16	−0.92	0.258
Ability to meet basic needs	−0.45	0.17	−0.48	−2.61	0.010

Older age, female gender and poorer access to camp services were associated with greater psychological distress at baseline. Participants who had been in a refugee camp for 1–2 years showed greater decreases in psychological distress over time than those who had been in a refugee camp for less than 1 year, or who had been in camps for 3 or more years. Furthermore, greater meeting of one's basic needs in refugee camps was associated with greater decreases in psychological distress over time.

## Discussion

To our knowledge, this is the first study to investigate the impact of displacement context on the course of psychological distress in refugees following resettlement. In this population-based study, we found that having previously lived in a refugee camp was associated with greater initial psychological distress for refugees after arrival in Australia, as well as faster decreases in psychological distress over the subsequent 4 years.

We start with interpretation of the higher initial psychological distress levels upon resettlement in Australia for those refugees who have lived in camps *v.* community settings. One possible explanation for this finding relates to differences in the displacement contexts and the resettlement environment in Australia. Specifically, it may be the case that the resettlement context in Australia was more similar to community contexts than camps, leading to lower initial distress amongst refugees who had previously lived in community settings after arrival in Australia. Although there is considerable variation in how refugee camps are organised, individuals living in camps are often dependent on humanitarian agencies for resources such as access to food, clean water, basic healthcare and face restrictions on engaging in economic activities and movement (Bakewell, 2003; Wright and Plasterer, 2010; UNHCR, 2014; SPHERE, 2018). In contrast, refugees who had previously lived in community settings may have been more accustomed to meeting basic needs and navigating the socio-economic context in a new country, and thus experience lower distress upon arrival in Australia than those who had previously lived in camp contexts. This explanation is supported by the finding that refugees who had previously lived in camps reported greater perceived stressors after arrival in Australia compared to those in non-camp environments, and that, after controlling for these stressors, there were no longer group differences in initial distress between those who had previously lived in camp relative to non-camp settings. This provides preliminary evidence that the initial navigation of the resettlement environment may be especially challenging for individuals who had previously lived in structured, camp-like settings. This explanation is speculative, and limited by the cross-sectional nature of the association between psychological distress and reporting of adjustment stressors for refugees recently arriving in Australia; for example, it is possible that greater psychological distress influenced reporting of resettlement stressors. However, we believe the finding of higher initial distress for refugees who have lived in camps requires further investigation to characterise the differences in initial adjustment for refugees resettling from camp relative to non-camp contexts.

A second key finding was that, after initial adjustment to the resettlement context, refugees who had previously lived in community settings showed slower psychological recovery than those who had lived in camp environments. One possible explanation for this finding is that refugees who had previously lived in community settings may have experienced relatively lower predictability and controllability in the transition environment as a function of their past experiences. Urban and rural community settings give rise to a number of stressors over which the individual has little or no control such as homelessness, destitution and hunger, as well as heightened risk of arbitrary arrest and detention (Campbell, 2006; Thomas *et al*., 2011; Crisp *et al*., 2012; Zetter and Ruadel, 2016; Im *et al*., 2017; Wachter *et al*., 2018; Logie *et al*., 2019; Kunpeuk *et al*., 2021), which may contribute to a lower sense of controllability of the external environment. It is possible that the sense of helplessness experienced by non-camp refugees as a result of these experiences contributed to slower psychological recovery due to deferring conditions of controllability and predictability. These findings accord with broader evidence that prior experiences of lack of control negatively impact subsequent stress responses (Bryant *et al*., 2014; Hancock and Bryant, 2018), and that controllability and predictability in the post-trauma environment are associated with lower psychological distress, including amongst refugees and civilian survivors of war (Basoglu *et al*., 2005; Le *et al*., 2018). In contrast, refugees living in camps typically have access to some camp-wide services facilitating the meeting of basic needs (e.g. regular access to food and shelter, access to emergency medical care), which may have fostered some sense of predictability and controllability. This is supported by the finding that greater meeting of one's basic needs in camps was associated with faster reductions in psychological distress in refugees who had previously lived in camps. It is important to note, however, that in camp environments, there are often high rates of interpersonal violence and significant security concerns that may negate a sense of control (Falb *et al*., 2013; Farhat *et al*., 2018). Furthermore, camp settings vary widely; participants in this study were predominantly from camp-like settings in Indonesia, Nepal, Iran and Malawi which may have afforded a relatively higher level of security and resources than other camp environments, such as large camps in Africa, Asia or the Middle East (Holzl, 2001; Saad, 2020). Further research investigating mechanisms underlying psychological adaptation could shed light on this finding; for example, by examining whether perceptions of control in the displacement context mediate reductions in psychological symptoms in refugees over time.

Findings from this study should be interpreted in the context of several limitations. First, the camp contexts in which refugees had lived was limited to camps in Iran, Indonesia, Malawi and Nepal. While these represent diverse settings, it is not clear how generalisable these results would be to refugees who have lived in camps in other locations globally. Furthermore, many individuals in this study likely lived in ‘camp-like settings’ rather than formal refugee camps, however we were unable to distinguish between these two groups given this detail was not collected in the current study. More refined categorisation of refugee camps would enhance the investigation of the association between displacement context and subsequent psychological distress in future studies. Second, this study did not measure characteristics of the non-camp context; accordingly, we are unable to determine specific conditions in the non-camp environment that were predictive of ongoing psychological distress. In addition, the measurement of camp characteristics was limited, with this study implementing a checklist-type approach to these experiences. Future research that comprehensively examines specific domains of facilities and resources, and refugees' experience of the quality of these services and specific camp regulations, may be useful in determining which domains are most important to address in order to facilitate psychological functioning. Third, we excluded refugees who had applied for asylum after reaching Australia. These individuals likely had experiences that differed substantially from the sample in this study (e.g. experienced perilous boat journeys, immigration detention, greater exposure to PTEs, insecure visa status). While the sample size of this group in this study was too small to undertake a multi-group analysis, future research should consider how displacement conditions for this group influence subsequent mental health, especially as psychological distress has been found to be especially high amongst refugees with insecure visa status (Momartin *et al*., 2006; Nickerson *et al*., 2019).

Findings from this study have potentially important implications for policy, programme design and service provision. Overall, results suggest that there may be different trajectories of psychological recovery in resettled refugees according to displacement context. This suggests that the displacement environment has an important effect on subsequent psychological functioning, and that programmes tailored to the circumstances of particular sub-groups of refugees who have lived in different transitory contexts may be warranted to enhance psychological outcomes. For example, it may be the case that the provision of psychological services for refugees who have lived in camps should be prioritised immediately after arrival in Australia, whereas those who have come from non-camp settings may require more sustained access to psychological services to facilitate recovery over time. In addition, we found that, amongst participants who had been in a refugee camp, poorer access to services was associated with greater initial distress after arrival in Australia, and greater ability to meet one's basic needs in camps was associated with faster decreases in psychological distress in the 4 years following resettlement. This has important implications for governments, NGOs and services supporting refugees in emergency or transition settings, suggesting that conditions in the displacement environment (whether it be camp or community settings) are critical in setting up patterns of recovery from adverse experiences long into the future. For example, by focusing on providing refugees in transitory settings with resources to meet their basic needs for shelter, food, water and medical care, and access to services to promote health and education, governments in resettlement countries may be fostering conditions that assist refugees to adapt effectively when they reach the host country.

In summary, this study represented the first investigation of the impact of displacement context on ongoing psychological distress in a representative sample of resettled refugees. Findings highlighted the critical role of displacement context in influencing subsequent psychological distress and the course of recovery for resettled refugees, and point to ongoing differential needs of refugees according to their displacement experiences.

## Supplementary material

For supplementary material accompanying this paper visit https://doi.org/10.1017/S2045796022000324. click here to view supplementary material

## Availability of data and materials

Data are publicly available upon application from Building a New Life in Australia (aifs.gov.au).
