# Supplementary material for: Impact of displacement context on psychological distress in refugees resettled in Australia: a longitudinal population-based study
Source: Epidemiol Psychiatr Sci. 2022 Jul 12;31:e51. doi: 10.1017/S2045796022000324 (PMC9281487; doi:10.1017/S2045796022000324)
Supplement: Supplementary file 1 [file S2045796022000324sup001.docx]

**Supplementary Materials**

Recruitment

Participants were recruited into the study based on the migrating unit (individual person, or group/family). Principal applicants for a humanitarian visa, granted between May and December 2013, aged 18 years or over, were invited to participate in the study. A principal applicant is the person on the visa application upon which the approval to immigrate was based ^1^. If the principal applicant provided consent, secondary applicants (i.e. other members of the migrating unit who were 15 years or older, and were residing with the principal applicant at the time of Wave 1 data collection) were invited to participate ^2^. The number of interviews completed with adult participants (aged 18 and over) who had come to Australia via an offshore resettlement pathway at each wave was as follows: Wave 1: 1,887; Wave 2 = 1,605; Wave 3 = 1,551; Wave 4 = 1,634; Wave 5 = 1,568 (retention = 83.1%).

Measures

***Exposure to Potentially Traumatic Events***

The number of pre-resettlement PTEs participants may have experienced was measured via a self-report questionnaire. Items were selected by the BNLA project team based on a scientific literature review, focus-groups and in-depth interviews with key stakeholders in the settlement sector ^3,4^. At Wave 1, participants were asked if they, or members of their family, had experienced (before coming to Australia); extreme living conditions (e.g. lack of food, water, shelter or medicine), war or other conflict, violence, imprisonment or kidnapping, political or religious persecution, natural disasters, and/or other traumatic events. Response options were Yes/No. Pre-migration trauma exposure was indexed as a count of the number of events (excluding the category “other”) participants endorsed (0 to 6).

**Refugee Camp-Related Variables**

*Assessment of Refugee Camp Status*

In terms of pre-settlement context, participants indicated whether they had ever spent time in a refugee camp (Yes/No) before they came to Australia, using the following definition of a refugee camp: *‘There are many refugee camps around the world. They are usually run by the United Nations and often house thousands of refugees who are waiting for their claims to be processed by the UNHCR’.* Participants who responded ‘yes’ to this question were categorized as having lived in a refugee camp; participants who answered ‘no’ to this question were categorized as those who had never lived in a refugee camp. This group likely constituted individuals living in both urban and rural general community settings.

*Assessment of length of time living in a refugee camp.*

We calculated a variable representing how long participants had resided in refugee camps overall. This was derived from participant responses to questions relating to how long they had resided in each individual refugee camp in which they had lived. Option responses for these variables comprised 1 = *less than 3 months*, 2 = *3-5 months*, 3 = *5-12 months,* 4 = *1-2 years*, 5 = *3-5 years*, 6 = *6-9 years* and 7 = *10+ years*. To create a variable approximating total time spent in refugee camps, the upper limit of each of these categories in months was calculated (e.g. 1 = 3 months, 2 = 5 months, 6 = 108 months), with the exception of 10 years where the lower limit was used due to the lack of further information (7 = 120 months). These were then summed together for each participant, and coded into a categorical total time variable where 0 = <1 year, 1 = 1-2 years, 2 = 3-10 years, 4 = 10+ years; these were converted into three dummy variables, with 0=< 1 year serving as the reference variable

Data analysis

To account for clustering within households, the analysis was conducted using the TYPE = COMPLEX command, with the household identification unit being used as the clustering variable. We estimated latent factors for the intercept, linear slope and quadratic slope. We compared a no-change (intercept-only) model with linear slope and quadratic slope models.

Next, we examined the extent to which covariates representing characteristics and experiences prior to arrival in Australia predicted intercept and slope; covariates investigated included having previously resided in a refugee camp (0= previously lived only in non-camp settings, 1 = previously lived in refugee camp), gender (0 = male, 1 = female), age, PTE exposure, educational status (dummy coded; no schooling (reference group), primary school, high school, tertiary), and region of birth (dummy coded; East/Central Africa/Oceania (reference group), North Africa/Middle East, Asia).

We then investigated change in psychological distress in the sub-group of the sample who had previously lived n camps. Similar to described above, the pattern of change in psychological distress over time was first modelled, with intercept-only, linear slope and quadratic models being tested. Next, covariates representing participant characteristics and experiences in camps prior to arrival in Australia were included in the model, comprising significant covariates from the first stage of analyses, in addition to key camp characteristics including number of camps lived in (ranging from 1-4), location of camps (four dichotomous variables; Iran, Nepal, Malawi, Indonesia), time in camp (dummy coded; less than 1 year, 1-5 years, 5+ years), having family in camp (0= no family in camp, 1 = at least some family in camp), lack of access to services in camps (count variable), available resources in camp (count variable). Notably, we omitted region of origin from this analysis as it strongly associated with camp location.

Results

Association between Refugee Camp Status and Perceived Stressors at each Timepoint.

We investigated whether participants who had lived in camps and non-camp settings differed in terms of perceived stressors experienced in Australia at each Wave. Participants who had lived in refugee camps reported significantly greater perceived stressors at baseline (M = 3.43, SD = 2.43) compared to those who had previously lived in community settings (M = 3.02, SD = 2.09, t(512.14) = 3.25, *p*=.003). In contrast, participants in refugee camps reported significantly lower perceived stressors at Waves 2 to 4 (but not Wave 5) compared to those who had previously lived in community settings (Wave 2 camp M = 2.28, SD = 1.36, community M = 2.73, SD = 1.45, t(983) = -4.10, *p*<.001; Wave 3 camp M = 2.81, SD = 1.98, non- community M = 2.73, SD = 1.45, t(1395) = -5.05, *p*<.001, Wave 4 camp M = 2.60, SD = 1.86, non- community M = 3.14, SD =2.00, t(915) = -3.41, *p*<.001, Wave 5 camp M = 1.91, SD = 1.78, non- community M = 2.14, SD = 1.77, t(1380) = -1.90, *p*=0.058).

Results of Conditional Latent Growth Curve Model of Change in Psychological Distress Over Time for Overall Sample, Controlling for Baseline Stressors

| Table. Predictors of baseline psychological distress and change in psychological distress over time in overall sample, controlling for baseline stressors | | | | | |
| --- | --- | --- | --- | --- | --- |
|  | B | S.E. | β | t | p |
| *Baseline psychological distress* |  |  |  |  |  |
| Previously lived in camp | 0.31 | 0.33 | 0.03 | 0.96 | 0.340 |
| Gender | 1.41 | 0.22 | 0.17 | 6.40 | <.001 |
| Age | 0.05 | 0.01 | 0.16 | 4.67 | <.001 |
| Baseline stressors | 0.69 | 0.06 | 0.36 | 11.24 | <.001 |
| Education |  |  |  |  |  |
| No education | - | - | - | - | - |
| Primary education | -0.64 | 0.40 | -0.06 | -1.60 | 0.109 |
| High school education | -0.40 | 0.39 | -0.05 | -1.04 | 0.296 |
| Tertiary education | -0.26 | 0.52 | -0.02 | -0.50 | 0.617 |
| Region of origin |  |  |  |  |  |
| Sub-Saharan Africa | - | - | - | - | - |
| Middle East/North Africa | 3.69 | 0.59 | 0.44 | 6.28 | <.001 |
| Asia | 1.44 | 0.58 | 0.17 | 2.49 | 0.013 |
| PTE exposure | 0.03 | 0.10 | 0.01 | 0.25 | 0.803 |
|  |  |  |  |  |  |
| *Change in psychological distress* | |  |  |  |  |
| Previously lived in camp | -0.57 | 0.13 | -0.26 | -4.54 | <.001 |
| Gender | -0.03 | 0.08 | -0.02 | -0.36 | 0.721 |
| Age | 0.00 | 0.00 | 0.05 | 0.96 | 0.337 |
| Baseline stressors | -0.09 | 0.02 | -0.23 | -4.45 | <.001 |
| Education |  |  |  |  |  |
| No education | - | - | - | - | - |
| Primary education | 0.10 | 0.15 | 0.04 | 0.63 | 0.527 |
| High school education | -0.07 | 0.14 | -0.04 | -0.53 | 0.598 |
| Tertiary education | -0.45 | 0.18 | -0.18 | -2.53 | 0.012 |
| Region of origin |  |  |  |  |  |
| Africa | - | - | - | - | - |
| Middle East/North Africa | -0.66 | 0.21 | -0.37 | -3.14 | 0.002 |
| Asia | -0.64 | 0.21 | -0.35 | -3.11 | 0.002 |
| PTE exposure | 0.04 | 0.04 | 0.06 | 1.15 | 0.252 |

In this model, female gender, older age, living in the Middle East/North Africa or Asia, and greater baseline stressors were associated with greater psychological distress at baseline. Having previously lived in a refugee camp was not associated with greater baseline distress. In this model, having previously lived in a refugee camp was associated with greater reductions in psychological distress over time, as were higher baseline stressors, tertiary education (compared to no education) and being from the Middle East/North Africa or Asia.

Results of Unconditional Latent Growth Curve Model of Change in Psychological Distress Over Time for Participants who had Previously Lived in Refugee Camps

Latent growth curve model analyses of changes in psychological distress over time in participants who had previously lived in refugee camps indicated that the model comprising intercept and linear slope (AIC = 9332, BIC = 9361, RMSEA = <.001, CFI = 1.00, TLI = 1.00) better fit the data than the intercept-only model (AIC= 9375, BIC = 9402, RMSEA = 0.09, CFI = 0.64 TLI = 0.72). The quadratic model failed to converge, and thus the linear-only model was retained. The linear slope model showed a significant intercept (B = 12.30, SE = 0.27, β = 4.31, p<.001) and negative slope (B = -0.59, SE = 0.10, β = -0.86, p<.001) suggesting that psychological distress was significantly greater than zero at baseline for this sample, and that, psychological distress decreased overall over the period of four years. There was no significant negative association between intercept and slope.

References

1. Australian Department of Social Services AIoFS, Colmar Brunton Social Research MMaM. Building a New Life in Australia: the longitudinal study of humanitarian migrants: Data User's Guide Release 4.0, 2018.

2. Edwards B, Smart D, De Maio J, Silbert M, Jenkinson R. Cohort Profile: Building a New Life in Australia (BNLA): the longitudinal study of humanitarian migrants. *International journal of epidemiology* 2017; **47**(1): 20-h.

3. Australian Department of Social Services AIoFS, Colmar Brunton Social Research MMaM. Building a New Life in Australia: the longitudinal study of humanitarian migrants: wave 1 data users guide. Australian Department of Social Services, Australian Institute of Family Studies, Colmar Brunton Social Research MMaM.: Australian Department of Social Services, Australian Institute of Family Studies, Colmar Brunton Social Research MMaM., 2015.

4. Khoo S-E. Key Research Questions for a Longitudinal Survey of Refugee and Other Humanitarian Migrants: Department of Immigration and Citizenship; 2012.
